# Performance enhancement in clustering cooperative spectrum sensing for cognitive radio network using metaheuristic algorithm

**DOI:** 10.1038/s41598-023-44032-7

**Published:** 2023-10-06

**Authors:** Vikas Srivastava, Parulpreet Singh, Shubham Mahajan, Amit Kant Pandit, Ahmad M. Alshamrani, Mohamed Abouhawwash

**Affiliations:** 1 Lovely Professional University, Phagwara, India; 2 Axis Institute of Technology and Management, Kanpur, India; 3 Hourani Center for Applied Scientific Research, Al-Ahliyya Amman University, Amman, Jordan; 4 University Centre for Research and Development (UCRD), Chandigarh University, Mohali, India; 5 Ajeenkya DY Patil University, Pune, India; 6 School of Electronics and Communication, Shri Mata Vaishno Devi University, Katra, 182320 India; 7 Department of Statistics and Operations Research, College of Science, King Saud University, P.O. Box 2455, 11451 Riyadh, Saudi Arabia; 8 Department of Computational Mathematics, Science and Engineering (CMSE), College of Engineering, Michigan State University, East Lansing, MI 48824 USA; 9 Department of Mathematics, Faculty of Science, Mansoura University, Mansoura, 35516 Egypt

**Keywords:** Energy science and technology, Engineering

## Abstract

Spectrum sensing describes, whether the spectrum is occupied or empty. Main objective of cognitive radio network (CRN) is to increase probability of detection (P_d_) and reduce probability of error (P_e_) for energy consumption. To reduce energy consumption, probability of detection should be increased. In cooperative spectrum sensing (CSS), all secondary users (SU) transmit their data to fusion center (FC) for final measurement according to the status of primary user (PU). Cluster should be used to overcome this problem and improve performance. In the clustering technique, all SUs are grouped into clusters on the basis of their similarity. In cluster technique, SU transfers their data to cluster head (CH) and CH transfers their combined data to FC. This paper proposes the detection performance optimization of CRN with a machine learning-based metaheuristic algorithm using clustering CSS technique. This article presents a hybrid support vector machine (SVM) and Red Deer Algorithm (RDA) algorithm named Hybrid SVM–RDA to identify spectrum gaps. Algorithm proposed in this work outperforms the computational complexity, an issue reported with various conventional cluster techniques. The proposed algorithm increases the probability of detection (up to 99%) and decreases the probability of error (up to 1%) at different parameters.

## Introduction

The fast growth of wireless communications has resulted in many new sorts of services or applications for humans. The proliferation of various communication equipment causes the public frequency band to become increasingly congested^1^.In the 5G communication system, the role of cognitive radio is essential. Cognitive radio senses and detects free channels and enhances the radio spectrum through opportunistic and dynamic access. CRN has two types of users: PU means licensed users, and SU means unlicensed users^2,3^. SU senses radio spectrum and recognizes channels that primary licensed users do not occupy at a particular location and time^4^. SUs can use spectrum holes to avoid interference with PU. Many spectrum sensing techniques have been used to detect the spectrum. Examples are energy detection, matched filter, Euclidean distance, autocorrelation, and cyclostationary feature detection^5–8^. One of the essential technologies in the CR system based on channel prediction and radio spectrum distribution is spectrum sensing. If spectrum detection accuracy is low, it may cause significant interference to primary network users, limiting the development and use of cognitive radio technology.

Sensing performance is affected by noise, multipath fading or shadowing, uncertainty, and spatial diversity. SU receiver sensitivity should be high enough to detect weak and noisy PU signals. For this, complex and expensive hardware is required. Costly and complex hardware requirements are the problem^9^. The solutions to these problems are CSS. CSS discusses the sensing efficiency of each SU. SUs share their information and take a joint decision that is beneficial for all SUs. Decision accuracy and sensing efficiency are increased by exploiting links among SUs in the same environmental area^10,11^. Many CSS techniques have been proposed. First is the distributed CSS technique, in which every SU exchanges its results with sensing spectrum^12^. The problem with this technique is that it requires considerable processing time to get the final decision. Second, the centralized CSS technique was proposed to overcome this problem, requiring an FC to send and receive data to and from SUs^13^ The FC is a key component of cognitive radio networks. By fusing the local sensing decisions of the SUs, the FC can make more accurate decisions about the presence or absence of PUs. This can help to improve the overall performance of the cognitive radio network. The FC in a cognitive radio network is a central node that collects local sensing decisions from SUs and fuses them to make a global decision about the presence or absence of a PU. The FC can be formulated in a number of ways, but the most common approach is to use a decision fusion rule. A decision fusion rule is a mathematical function that takes as input the local sensing decisions of the SUs and outputs a global decision. There are many different decision fusion rules like AND rules, OR rules, and Majority rules. The choice of decision fusion rule depends on the application. For example, if the application requires a very low false alarm rate, then the AND rule may be a good choice. If the application is more tolerant of false alarms, then the OR rule or the majority rule may be a better choice. In addition to the decision fusion rule, the FC can also use a number of other parameters to fuse the local sensing decisions of the SUs. These parameters include are weights and threshold. The choice of weights and threshold depends on the application and the desired performance. In terms of detection rate, CSS is better than non-cooperative spectrum sensing. The disadvantages of CSS are extra power consumption, system overhead, more significant data exchange, and higher processing time. To overcome these problems, an advanced FC requires the processing of high-level computational signals. The channel state is determined based on sensing data forwarded by SUs to the FC. The requirement for an FC produces high computational capability. To overcome this problem, we use cluster-based spectrum sensing. In cluster, SUs transmit their data to cluster head. In the traditional energy detection method, SUs compare received signal results with the prior threshold and decide. If noise exists, the threshold is mismatched with the correct threshold, and the FC gets the wrong result. The author in^14^ discussed CSS based on dual-threshold to remove the limitation of threshold mismatch in energy detection. Here the author used credibility metric for sensing the results of SUs. These reliable sensing results lie in the decision zone. So, in^14^, the author discussed the dynamic dual-threshold CSS technique to enhance detection performance and remove sensing breakdown problems under noise power uncertainty. No detection occurs when energy falls between two thresholds, resulting in poor performance, such as a decrease P_d_ and a longer spectrum sensing time. To solve these issues, adaptive double threshold CSS using historical energy detection was suggested to solve them^15^. It is shown in^16^ that calculates the optimum threshold level value for improving spectrum sensing performance while maintaining the lowest possible error probability. To minimize the combined impacts of noise uncertainty and asynchronous primary user occurrences within the sensing period of the SU in a heterogeneous cognitive network, it is necessary first to identify the SU's sensing interval.

The article^17^ attempts to minimize the combined impacts of noise uncertainty and asynchronous primary user occurrences within the SU's sensing period in a heterogeneous cognitive network. So, as a result, an asymmetrical scale sampling criterion-based double threshold energy detection technique was suggested. A CRN suggested the greedy algorithm and particle swarm optimization to improve spectrum sensing^18^. In^19^, mathematical modelling and critical assessment of detection probability for energy detection-based spectrum sensing at low SNR in an uncertain, noisy environment are presented. A mathematical model has been suggested to calculate two thresholds for reliable sensing when measured energy is smaller than noise power uncertainty.

Here, a cluster-based CSS scheme is proposed. It depends on the cluster technique to increase performance of CRNs by increasing the probability of detection and decreasing probability of error with help of a machine learning-based metaheuristic algorithm. The probability of error is a combination of the probability of missed detection and the probability of false alarm. Clustering is a process of dividing a large network into smaller groups, or clusters, in order to improve performance and scalability. In CRNs, clustering is often used to form a virtual backbone, which can help to reduce communication overhead and improve the overall efficiency of the network. There are a number of different ways to create clusters in CRNs. One common approach is to use a centralized algorithm, in which a central node is responsible for assigning nodes to clusters. Another approach is to use a distributed algorithm, in which nodes themselves are responsible for selecting their own CHs.

Once clusters have been created, they need to be maintained in order to ensure that they continue to function properly. This involves tasks such as keeping track of the cluster members, electing new CH if necessary, and resolving conflicts between clusters. If the CH of a cluster becomes unavailable, then the cluster needs to be re-elected. This can be done using a number of different methods, such as a round-robin election or a voting algorithm. The key considerations for cluster creation, maintenance, and re-election in CRN are performance, scalability, robustness and security. Benefits of clustering in CRNs are scalability, efficiency and robustness. This paper proposed a novel combination of SVM and the RDA.

The organization of this paper is as follows: “Related work and problem formulation” Section provides related work regarding cluster-based CSS techniques and problem formulation. “Theoretical background” Section describes the theoretical foundation. “Proposed SVM–RDA Algorithm” Section describes conventional RDA and SVM algorithms. “Proposed methodology” Section describes methodology. “Results” Section discusses simulation results and compares the proposed technology with other technologies. “Performance analysis” Section describe performance analysis. Finally, the conclusion is part of “Conclusion” Section.

## Related work and problem formulation

One of most challenging issues facing CRN is spectrum sensing^20^. An enormous amount of scientific interest has been generated. The literature has developed several narrowband spectrum sensing methods based on matched filtering, energy detection^5^, and cyclostationary feature detection^8^. To address the inaccuracies of the sensing technique, multiple detection thresholds should be used. For example,^21^ suggested energy detection with 2 thresholds to increase sensing decision accuracy. That is called CSS. CSS has two types, i.e., centralized and distributed. Forwarding sensing information to an FC is called centralized spectrum sensing^21^, and distributed spectrum sensing involves nodes sharing information among themselves to form a decentralized decision about spectrum holes^22^. Multiband joint detection^23^ and wavelet-based spectrum sensing^24^ are techniques to improve the performance of narrowband techniques across a broad bandwidth. Several methods have been suggested to reduce the need for such a high sample rate. As a result, several narrowband sensors detect the wideband at the same time.

In CRN, illegal SU access free permitted channels while there is less interference with PU and other SU^25^. The SUs are equipped with an unexpected and dynamic environment^26^. Energy detection methodology is considered due to its simplicity and low computational cost. For detection of PU, use proper energy threshold^27^. The author of^28^ investigates the impact of energy detection, CSS on CRN, and threshold selection in a fading atmosphere. Umebayashi^29^ presented a hierarchical CSS method using a dual-threshold energy detection mechanism. The author^30^ investigates a device to devise communication that combines 2 secondary links, 1 primary link, and a relay network and discusses the cluster relay selection method for secondary and primary transmission. Unfortunately, L1 norm minimization is unsuitable and mathematically expensive for many real-time applications. Threshold optimization matching pursuit (TOMP) is time-consuming due to the large number of projections. Cooperative sensing in a wireless environment reduces shadow and multipath fading effects^31^. In^32^, with help of TOMP algorithm, the sparse wideband spectrum signals are reconstructed^33,34^. With use of wavelet-based edge detector, boundaries between spectrum bands are estimated^24^ originally introduced in^35^. Fast matching pursuit (FMP) is an accurate and fast threshold-based greedy recovery algorithm for compressed sensing. The main aim of fast matching pursuit is to reconstruct a sparse signal from samples collected at a much lower rate than the Nyquist rate, as accurate and fast as possible, and apply it in a wide range of CRN applications. Unlike related works, FMP exploits the spectrum signal in the wavelet packet domains in which the spectrum is sparser. The L1 norm wavelet packet (WP) is also a greedy recovery algorithm^36^.

The author in^37^ describes the formation of a cluster of secondary users based on artificial intelligence based on machine learning and compares performance in terms of energy efficiency. The author in^38^ discussed CSS techniques with low complexity to get high cooperative gain and increase sensing results. The performance of existing techniques is good, and these techniques can remove uncertainty due to multipath fading and shadowing, and allowing for easy signal recovery with a satisfactory detection rate. The disadvantage is a low probability of detection and a high probability of error. In^39^, multiple reporting channels (MRC) for cluster-based cooperative CRN are proposed, which allows for greater use of the reporting time frame by increasing the sensing time of SU, thus reducing the overall cost.

Wideband spectrum sensing is available on several channels with several frequency bands. For wideband spectrum sensing, compressive spectrum sensing has been proposed^40,41^. In^36^, the author discussed centralized CSS, where autocorrelation results of SU’s signal are transferred to an FC for decision making. At the FC, these signals are recovered by a different matching pursuit recovery algorithm. These matching pursuits’ algorithms introduce fast and accurate spectrum sensing techniques.

Attributes of cooperative sensing are sensing, transmission and reporting^42^. When total number of SU increases in cooperative sensing, energy consumption also increases. So Probability of detection (P_d_) reduces and probability of error (P_e_) increases. To overcome this problem, SUs are grouped into different sets based on their sensing results. After that, P_d_ increases and P_e_ decreases at different parameters like signal to noise ratio, number of secondary users, and number of occupied bands. In earlier literature, SUs were grouped randomly or based on received signal strength. In earlier conventional clustering method, SU senses the result and CH is far from cluster and near to FC. Nodes in a cluster are close to each other but different from nodes of other clusters. Clustering is an unsupervised machine learning technique that groups data points together based on their similarity. Data points within the same cluster are more similar to each other than they are to data points in other clusters. So objective is to increase performance of clustering CSS with increase probability of detection and decrease probability of error by machine learning-based metaheuristic algorithm or learn heuristic algorithm. With learn heuristic algorithm, they select best SU from CRN, which saves energy consumption and partitions SUs into cluster. In spectrum sensing, P_d_ should be high to reduce disturbance due to PU and P_e_ should be low to increase spectrum utilization.

## Theoretical background

In contrast to the cooperative SUs in the network, each SU conducts local spectrum sensing in isolation. The signal received by the jth SU is:

1 $$x_{j} = h_{j} *s + n_{i} H_{1}$$

2 $$x_{j} = n_{j} H_{0}$$

$x_{j}$ is signal received by jth SU, $h_{j}$ is channel gain between jth SU and PU, s is the signal of PU, and n_i_ is noise. Hypothesis H_1_is existence of PU and hypothesis H_0_ is absence of PU. An FC performs the final spectrum sensing and coordination amongst collaborating SUs for large clusters of SU. It may be accomplished via the use of two methods. In first approach, collaborating SUs run local spectrum sensing independently to find results from the sensors. Consequently, they send their local findings to the FC, making the ultimate judgments through reporting channels. FC integrates incoming data and determines whether a PU signal is present in the detected channel. FC communicates absolute judgment to all SUs. According to second approach, cooperating SUs transmit their captured data to FC, responsible for performing spectrum sensing.

Individual spectrum sensing performance is used to calculate CSS performance. The probability of detection P_d_, the probability of false alarm P_fa_, and the probability of missed detection P_md_ are the metrics that are being measured. P_d_ is the probability of detection, which means an SU announces existence of a PU signal while spectrum is occupied. The probability of detection is

3 $$P_{d} = Prob\left( {\frac{{H_{1} }}{{H_{1} }}} \right)$$

H_1_ is a binary hypothesis linked to the existence of the PU signal. Individual P_fa_, is defined as probability that SU reports existence of PU signal with clear spectrum. The probability of false alarm is.

4 $$P_{fa} = Prob\left( {\frac{{H_{1} }}{{H_{0} }}} \right)$$

H_0_ is a binary hypothesis linked to the absence of the PU signal. P_md_ is probability that SU announces absence of PU signal while PU occupies spectrum. The probability of missed detection is

5 $$P_{md} = Prob\left( {\frac{{H_{0} }}{{H_{1} }}} \right)$$

C_d_ is the cooperative probability of detection.

6 $$C_{d} = 1 - \left( {1 - P_{d} } \right)^{L}$$

P_d_ is the probability of detection, and L is number of cooperating SU. Probability of error (P_e_) is a combination of P_md_ and P_fa_.

7 $$P_{e} = P_{md} + P_{fa}$$

CSS methods provide reasonable detection rates. However, CSS functions effectively with small SUs density. With a large density of cooperating SUs, these methods are complicated and slow to process. As a result^43–49^, suggested a clustering method based on CSS. SUs are clustered according to specific characteristics, (i) geographic region and (ii) distance between PU and SU. One SU serves as cluster head (CH) for each cluster, coordinating communications between its users and FC. At every cluster, SUs carry out their spectrum sensing individually and then transmit their findings to CH via a network of communication links. The findings are sent to the FC, where final decisions are made by the CH^45^. Individual spectrum sensing clustering performance metrics are also used for CSS^47^.Due to the analog to digital converter requirement, Nyquist sampling rate is not valuable for wideband spectrum sensing. So the author in^50^ reviews wideband spectrum sensing based on Nyquist sampling.

SUs in conventional energy detecting technologies often make decisions by comparing incoming signals to a previous threshold^51^. H_0_ or H_1_ is selected depending on whether received signal strength of PU is more than or less than threshold^52^. However, when SNR is low, detection effectiveness of conventional energy detection methods suffers significantly. An adaptive dual threshold energy detector was suggested as a solution to the issue of noise uncertainty, and it was developed using the best single threshold value. The author in^53^ describes CSS and non-CSS for full-duplex on a time-selective Nakagami-m fading channel.

## Proposed SVM–RDA algorithm

### Red deer algorithm

The RDA begins with a random population, similar to how other meta-heuristics work. A few of the finest RDs from among the population are chosen and designated as the ‘male RD,” while the other RDs are referred to as "hinds.' First and foremost, the male RD must roar. They are classified into two categories based on roaring power (i.e., stags and commanders). “After that, stags and each harem's commanders fight together to take control of their harem. Number of hinds in harems is proportional to commanders' fighting and roaring skills. As a result, commanders form harems where they mate with large numbers of hinds. Other male stags pair with their closest hinds regardless of the harem's size restriction. However, we only accept solutions that are better observed throughout the fighting between commanders and stags; in a similar way. Afterwards, harems are created and distributed among the commanders according to their degree of power. While doing the exploration phase, this step aids the algorithm. As a result, a harem commander mates with α percentage of hinds from his harem and β percentage of hinds from another harem. All stags should mate with closest hind during breeding season, i.e. they should only consider the distance between them and the hind and not the harem's restrictions. This stage considers both the exploration and exploitation phases at the same time”^54^.

#### Initialize red deer

Another critical stage of the RDA is the mating process, which results in the generation of RD progeny. The objective of enhancement is to develop a solution that is close to global in terms of problem’s variables. In machine learning, an array of RDA means red deer, and an array is:

8 $${\text{Red}}\,\,{\text{Deer}}\, = \,\left[ {{\text{X}}_{{1}} ,\,{\text{X}}_{{2}} ,\,{\text{X}}_{{3}} , \ldots ;{\text{X}}_{{{\text{Nvar}}}} } \right]$$

To begin the process, we create an N_pop_ starting population. We assign N_male_ to a subset of the best RDs and N_hind_ to the remainder (N_hind_ = N_pop_ − N_male_).

#### Roaring male RD

The male RD is attempting to enhance their elegance by screaming at this step. As a result, the roaring process may succeed or fail, just as it does in nature. Interestingly, in this methodology, male RDs come out on top regarding the available solution set. If the objective functions of neighbors are better than male RD, replace them with the prior ones. Permit every male RD to change their position. To update the position of males, the following equation is proposed:

9 $$male_{new} = \left\{ {\begin{array}{*{20}c} {male_{old} + a_{1} X \left( {\left( {UB - LB} \right)*a_{2} } \right) + LB if a_{3} > 0.5} \\ {male_{old} - a_{1} X \left( {\left( {UB - LB} \right)*a_{2} } \right) + LB if a_{3} < 0.5} \\ \end{array} } \right.$$

UB is upper bounds of search space, and LB is lower bounds of search space. Male_old_ is present position of the male RD, and male_new_ is its new position. a_1_, a_2_ and a_3_ lie between 0 and 1.

#### Appoint γ percentage of the best male RDs as male commanders

There is a wide range of differences amongst male RDs in nature. Some of them are stronger, more enticing, or better at extending their area than the rest of them. In this way, RD is subdivided into commanders and stags, each serving a distinct purpose. The following formula is used to measure the number of commander males

10 $$N_{com} = round \{ {{\varvec{\upgamma}}}_{*} N_{male} \}$$

N_Com_ is a number of male commanders. Finally, stags are counted as follows:

11 $$N_{slag} = N_{male} - N_{com}$$

N_Stag_ is the number of stags.

#### A battle between stags and male commanders

Assign stags to each commander at random. Two mathematical formulae for fighting are given by:

12 $$New 1 = \frac{Com + Stag}{2} + b_{1} *\left( {\left( {UB - LB} \right)*b_{2} } \right) + LB)$$

13 $$New 2 = \frac{Com + Stag}{2} - b_{1} *\left( {\left( {UB - LB} \right)*b_{2} } \right) + LB)$$

Suppose New1 and New2 are two newly produced solutions as a result of the fighting process. Com is commander, and the stag is stag. During the fight, a commander and a stag pursue one another. The result is the creation of two new solutions. One gets to choose the winner, while the other is out of the running.

#### Make harems

The commander creates harems here. A male commander captured a harem of hinds, and male commanders' strength determines harem size. To create the harems, we distribute hinds among commanders in a quantifiable way.

14 $$V_{n} = v_{n} - max_{i} \left\{ {v_{i} } \right\}$$

$v_{n}$ is the power of the nth commander. $V_{n}$ is a normalized value

Overall, more hinds go to the commander because of his higher fitness value.

#### Mate harem’s commander with ${\varvec{\alpha}}$ percentage of hinds in his harem

Deer reproduce in the same way that other animals do. A commander does this, and the parents make up a certain amount of the hinds in his harem.

15 $$N.harem_{n}^{male} = round\left\{ {\alpha .N.harem_{n} } \right\}$$

$N.harem_{n}^{male}$ is number of hinds from nth harem who mate with commander. Starting value of the RDA model's parameter is $\alpha$, which lies between 0 and 1. The formula of the matting process is:

16 $$offs = \frac{Com + Hind}{2} + \left( {UB - LB} \right)*c$$

Offs is a new solution. Value of c lies between 0 and 1.

#### Pairing a harem’s commander in another harem with β % of hinds

In this case, male commander mates with β % of the hinds in his harem. To extend his area, the commander attacked another harem. An initial parameter is β, which lies between 0 and 1. The commander's harem has the following number of hinds:

17 $$N.harem_{k}^{male} = round\left\{ {{\upbeta }.N.harem_{k} } \right\}$$

$N.harem_{k}^{male}$ is number of female red deer (hinds) from the kth harem who mate with the commander.

#### The stag pairs up with the closest hind

Each stag mates with its closest female red deer at this stage, and the male RD likes to track the female red deer during mating season. Without taking harem areas into account, this hind could be his favorites among all hinds. Stags were allowed to breed with hinds that were closest to them. People who are looking for a distance in j-dimension space should use the following formula to figure out which hind is closest:

18 $$d_{i} = \left( {\mathop \sum \limits_{j \odot J} \left( {stag_{j} - hind_{j}^{i} } \right)^{2} } \right)^{{{1 \mathord{\left/ {\vphantom {1 2}} \right. \kern-0pt} 2}}}$$

$d_{i}$ is the distance between ith hind and the stag.

### Support vector machine

A supervised machine learning technique known as the SVM may be utilised for classification and regression problems. But it is mainly used in categorization. The SVM algorithm's objective is to construct the optimal decision boundary or line that can divide n-dimensional space into classes so that subsequent data points may be conveniently placed in the correct category. A hyperplane denotes the optimum decision boundary. After that, categorization is carried out by locating the hyper-plane that sharply defines the two groups. Hyperplane position and orientation are affected by support vectors that are near the hyperplane. Here, optimize the classifier's margin using these vectors. The location of the hyperplane varies if the support vectors are removed. These are the aspects that assist us in developing our SVM^55^.

### Support vector machine-red deer algorithm

There are several meta-heuristic optimization algorithms developed that are inspired by nature. The efficiency of classification and prediction is improved by optimizing machine learning using the metaheuristic optimization algorithms^56–60^. Efforts have been made to improve SVM performance, but very few of these efforts have focused on SVM convergence. This study proposes a novel SVM–RDA algorithm. Flowchart of the proposed method is shown in Fig. 2. Proposed algorithm primarily comprises 2 procedures: (i) internal parameter optimization and the external classification performance evaluation. During the internal parameter optimization procedure, the SVM parameters are dynamically adjusted by the RDA method. The proposed SVM–RDA divides entire algorithm into two sub-sections. One section is demonstrated by SVM to initialize weight parameters and the other by RDA, in which the weight parameters are updated to find the best weight parameter value. The populations in RDA are randomly initialized. This proposed SVM–RDA included the benefits of a RDA for CH selection and energy-aware cluster formation. Machine learning, features extraction, regression, and classification operations are built on optimum parameters selection. In this paper, the RDA, a recent population-based meta-heuristic algorithm, is thoroughly reviewed. The RD algorithm combines the survival of the fittest principle from the evolutionary algorithms and the productivity and richness of heuristic search techniques.

The main steps conducted by the SVM–RDA are:

- Step 1-Create a system model using PU, SU, and FC. Generate a full-duplex multiband signal.
- Step 2-Check the availability of PUs in spectrums and collect FC, the energy value submitted by SUs in each round. Sense the availability of spectrum using the SVM algorithm.
- Step 3-*Initialise* the parameters of SVM such as bias and weight vector (training).
- Step 4: Make an SVM model that takes into account the energy level of the SU and the availability of the PU.
- Step 5: Run a spectrum sensing test on the network after training it with a low starting weightand put it through a spectrum sensing test. If not correctly sense the spectrum and then update the weight of SVM using the RDO algorithm.
- Step 6: Initialize the weight parameters of the red deer algorithm.
- Step 7: Roar, male red deer.
- Step 8: Select γ percentage of the best male red deer as the male commander.
- Step 9: The battle between the male commander and the stag and the formation of haram.
- Step 10: Mate commander with α and β % of hinds in another haram.
- Step 11: Mating the stag with the nearest hind and calculating a new weight parameter.
- Step 12: If this new weight parameter is not satisfied for spectrum sensing, then go to step 7.
- Step 13: If end criteria are satisfied, then SVM classification is based on optimum weight value as testing set.
- Step 14: It gives information about the spectrum that is available or not available.
- Step 15: Assign SU to the available spectrum.

## Proposed methodology

An FC and N SUs are considered in CSS, and the FC is in charge of all cooperative cognitive user channel allocation and monitoring. The suggested system acquires the SU received signal via a learn heuristic algorithm, a mix of machine and metaheuristic algorithms. Each SU transmits its signal to the CH, which recovers signal and senses spectrum from the data of the SUs. The CH then forwards their local choices to an FC, making the final decision on spectrum occupancy. All cooperating SUs transmit signal to CH for spectrum sensing. CH enables each SU to conduct its spectrum detection in the shortest amount of time possible, and then each SU sends its findings to the FC. As a result, our suggested method has a high P_d_ and a low P_e_ at different SNR and occupied bands. The FC in a cognitive radio network can be formulated for machine learning based metaheuristic algorithm in a number of ways. One approach is to use a decision fusion rule that is based on a machine learning algorithm. For example, a support vector machine (SVM) can be used to learn a decision boundary that separates the "busy" and "idle" states of the spectrum. The FC can then use this decision boundary to make a global decision about the presence or absence of a PU. Another approach is to use a metaheuristic algorithm to optimize the parameters of the fusion center. For example, RDA can be used to optimize the weights and threshold of the fusion center. The RDA can be used to search for a set of parameters that minimizes the false alarm rate and maximizes the detection rate.

### System model

Consider a CRN with N SU and M PU and central FC that is subject to Rayleigh fading effect. Same set of SU is grouped in a cluster. The SNR of each SU measure is compared to the threshold. If evaluated SNR is less than predefined threshold of SNR, SU is not allowed for cooperative sensing. While SNR of SU is greater than threshold, SU is selected for cooperative sensing.

**Figure 1:**
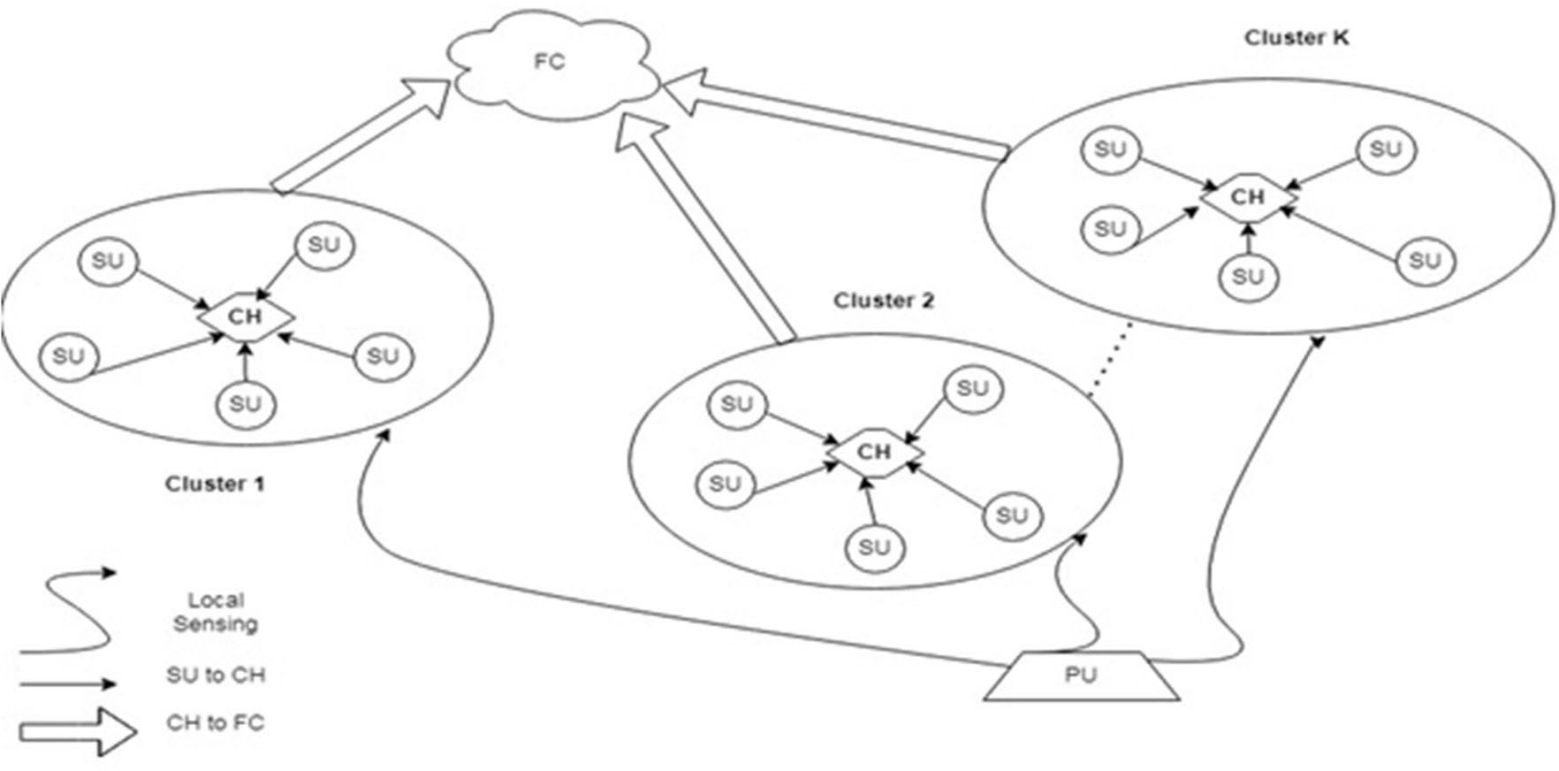
System model of clustering cooperative spectrum sensing.

Assume N SUs are chosen for a particular sensing phase. So number of SU in single cluster is D = N/K. N is no. of SU that makes cluster. As shown in Fig. 1, CH is one of the SUs from the cluster. Clustering CSS has two types: inter-cluster CSS and intra-cluster CSS. In intra cluster CSS, SU detects the presence of PU information and transmits this information to the corresponding CH, then CH makes a final decision related to that cluster and forwards the information to FC. In inter-cluster CSS, all CH transmit their information to the FC, and FC make final decision related to data of CH. Intra cluster CSS occurs between two clusters. Cluster decisions may not be accurate at times due to a weak PU received signal. So there is uncertainty about a single cluster decision. So, inter cluster CSS can overcome this problem in which combined decision is taken by different clusters. The CH of each cluster is selected using a machine learning-based metaheuristic algorithm among set of selected SU. Sort the best SUs into clusters. If there are more than one licensed channel, apply hierarchical clustering methods to group them into cluster! based on high similarity between them^61–64^. These cluster can communicate outside of the cluster with the help of Improved Hierarchical Clustering Algorithm (IHCA).

### Flowchart

**Figure 2:**
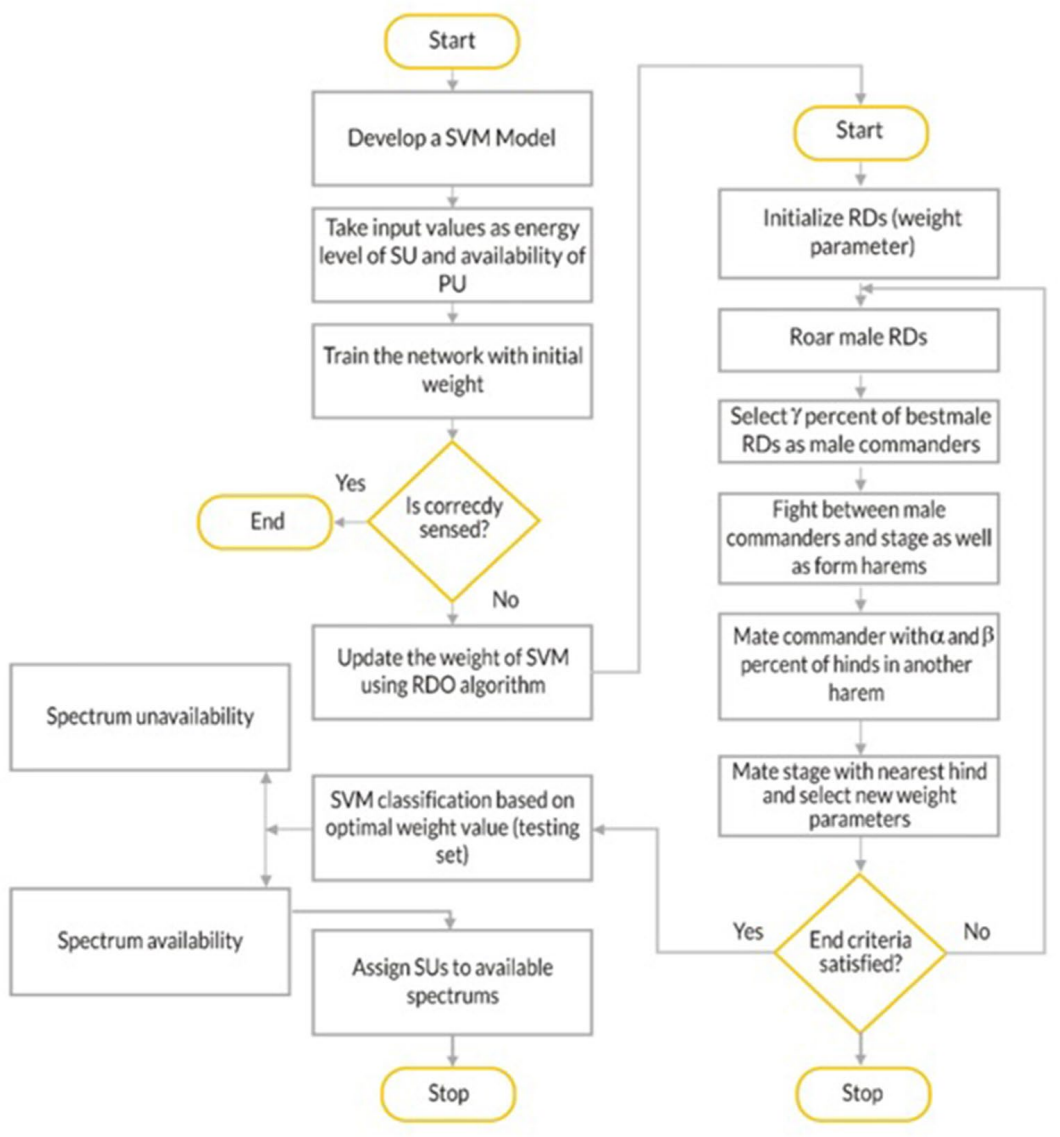
Flow chart of SVM–RDA.

A flowchart describing workflow of proposed SVM–RDA algorithm is shown in Fig. 2.

Pseudo code of SVM–RDA algorithm

1. Define the parameters:

- Number of clusters (K).
- Clustering algorithm (e.g., k-means, hierarchical clustering).
- SVM kernel (e.g., linear, radial basis function).
- SVM regularization parameter (C).
- SVM convergence criterion (e.g., maximum number of iterations).
- Spectrum sensing threshold (T).

2. Collect the spectrum data from the radio frequency (RF) sensors.
3. Perform clustering on the spectrum data to identify the clusters:

- Apply the chosen clustering algorithm to the spectrum data.
- Determine the cluster centers and assign each data point to the nearest cluster.

4. Initialize SVM for each cluster:

- For each cluster, create an SVM model with the chosen kernel and regularization parameter.

5. Train the SVM models:

- For each cluster, use the data points belonging to that cluster to train the SVM model.
- Set the class labels of the data points in the cluster to be 1 (occupied) if the signal strength is above the threshold T, and -1 (unoccupied) otherwise.

6. Spectrum sensing:

- Collect real-time spectrum data from the RF sensors.
- For each data point, determine which cluster it belongs to based on its proximity to the cluster centers.
- Use the corresponding SVM model for that cluster to classify the data point as occupied or unoccupied.
- If the majority of SVM models classify the data point as occupied, then consider the spectrum as occupied; otherwise, consider it unoccupied.

7. Repeat the spectrum sensing process periodically to adapt to dynamic spectrum usage.
8. End.

RDA is a nature-inspired optimization algorithm inspired by the behavior of RD. It is used for feature selection and optimization. In the above pseudo-code, we are combining the RDA with SVM for spectrum sensing, but the specific implementation of the RDA might vary depending on the context and the problem you are trying to solve. Additionally, the choice of clustering algorithm, SVM parameters, and other settings may need to be tailored according to your specific application and data. RDA balance between exploration (global search) and exploitation (local search) of the solution space. Unlike traditional optimization methods, RDA often require minimal problem-specific knowledge or parameter tuning^65^.

### Ethical approval

None of the authors’ experimented with human subjects or animals during this research.

## Result

**Figure 3:**
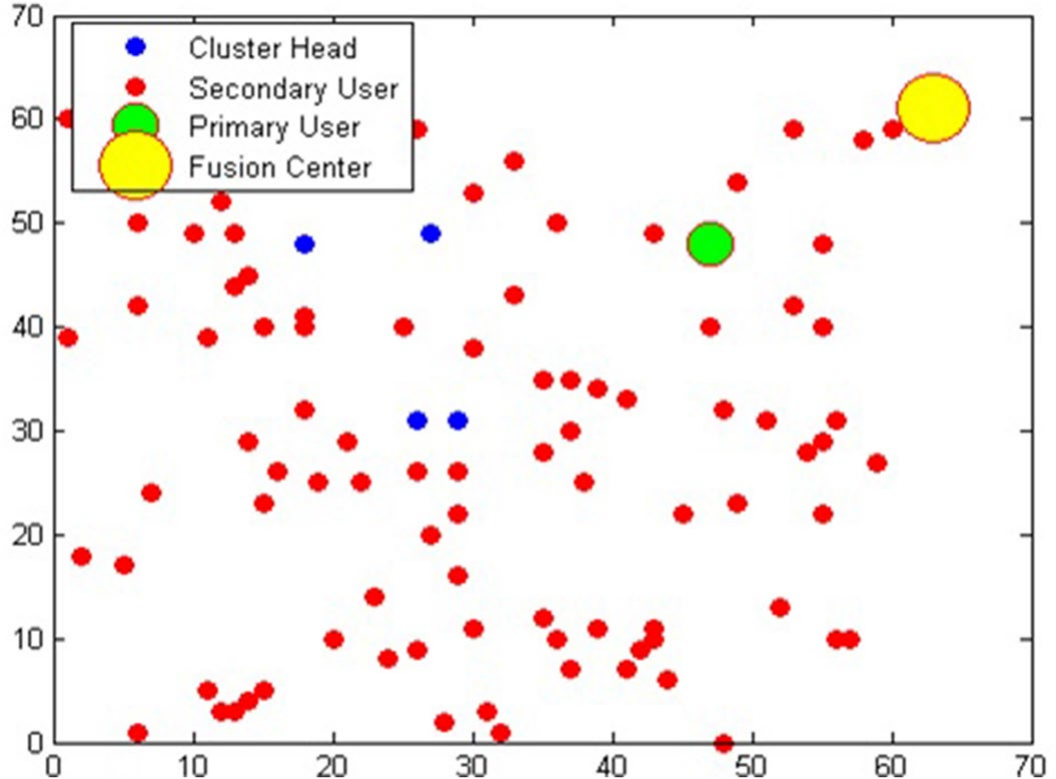
shows the positions of the PU, SU, FC and CH.

The suggested algorithm's performance is assessed using MATLAB R2014a on a 64-bit, core i5 processor, and 4 GB RAM system. The range of SNR is varied from − 20 to 20 dB. p(H0) = p(H1) = 0.5 are the probabilities of the PU being idle or busy state. The signal bandwidth is 7.56 MHz, and it is broadcast on the 720 MHz central radio frequency. The simulation results have been employed to show the dependency of P_d_ and P_e_ of CRN on SNR and occupied band. The probability of error is a combination of the P_fa_ and the P_md_. Here, analysis of the P_d_ and probability of error is based on SNR and the number of occupied bands using MATLAB. Improvements in the detection rates of CSS systems have shown acceptable results. However, they only need a small number of SUs to work effectively. This method is less effective because of the increased complexity and processing time required for many cooperating SUs. As a result, an FC solution based on CSS was suggested. SU is coordinated with the FC. As a result, SUs perform independent spectrum sensing and transmit their combined results to the FC for final decision. Figure 3 represents an FC-based CSS. Suppose that there is 1 PU and 90 SUs randomly dispersed in a square field with a length of 70 m. Here, PU uses the free-space path loss model. Figure 3 shows PU, SU, CH, and FC are distributed over 70 × 70 m.

**Figure 4:**
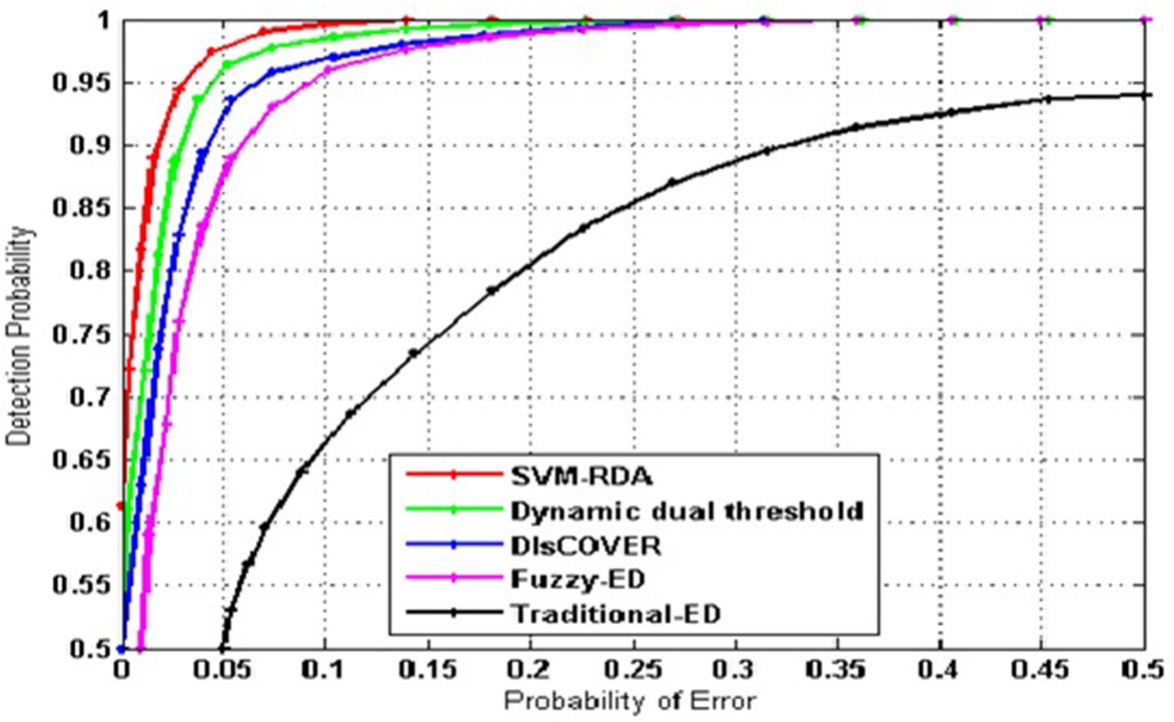
Probability of detection vs Probability of error.

The performance of SVM–RDA algorithm is compared with DIsCOVER, Fuzzy-ED, Traditional-ED, and dynamic dual-threshold. Figure 4 represents the P_d_ versus P_fa_ with noise uncertainty under -12 dB SNR values. Because SUs have a low Pfa, they can easily reach approved bands that aren't being used. This means that more spectrum resources can be used by a lot. Decreasing P_d_ means PU is disturbed by SUs. Under low noise uncertainty, the detection performance of traditional-ED decreases. By comparing, the proposed algorithm can show strong performance in detection in the worst case. As a result, it can achieve a higher P_d_ than other methods under same P_fa_. In our proposed method (SVM–RDA), the P_d_ is significant, and the P_e_ is less.

**Figure 5:**
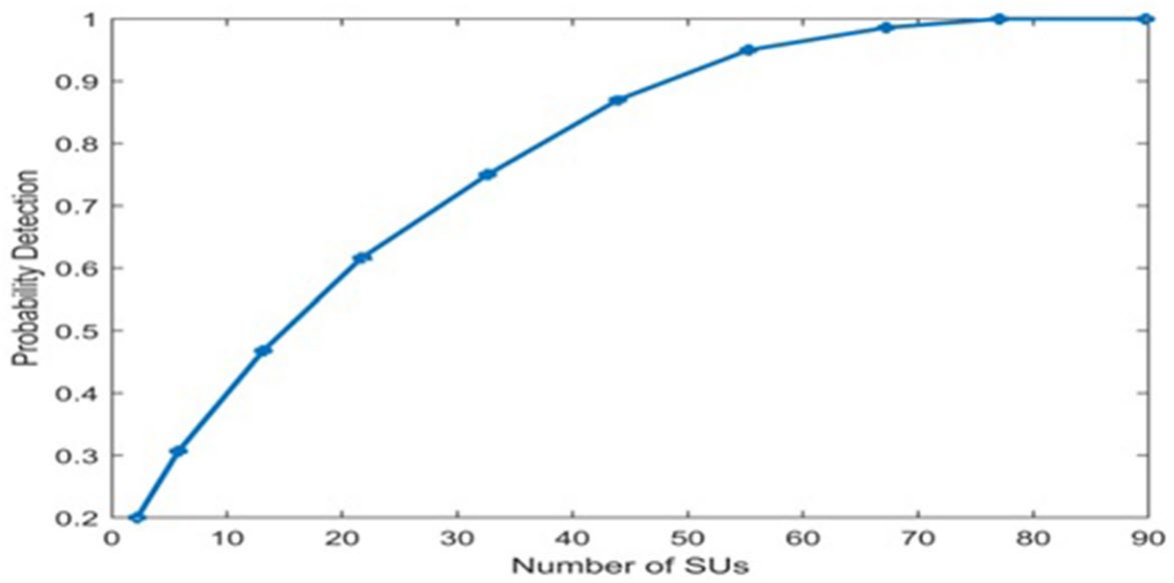
Probability of detection versus no. of cooperative SU.

Figure 5 describes P_d_, versus number of cooperating SUs. As seen below, the P_d_ rises directly proportional to the number of collaborating SUs. So, detection rate rises as number of SUs grows, and detection performance improves means P_d_ will increase, that give good performance.

**Figure 6:**
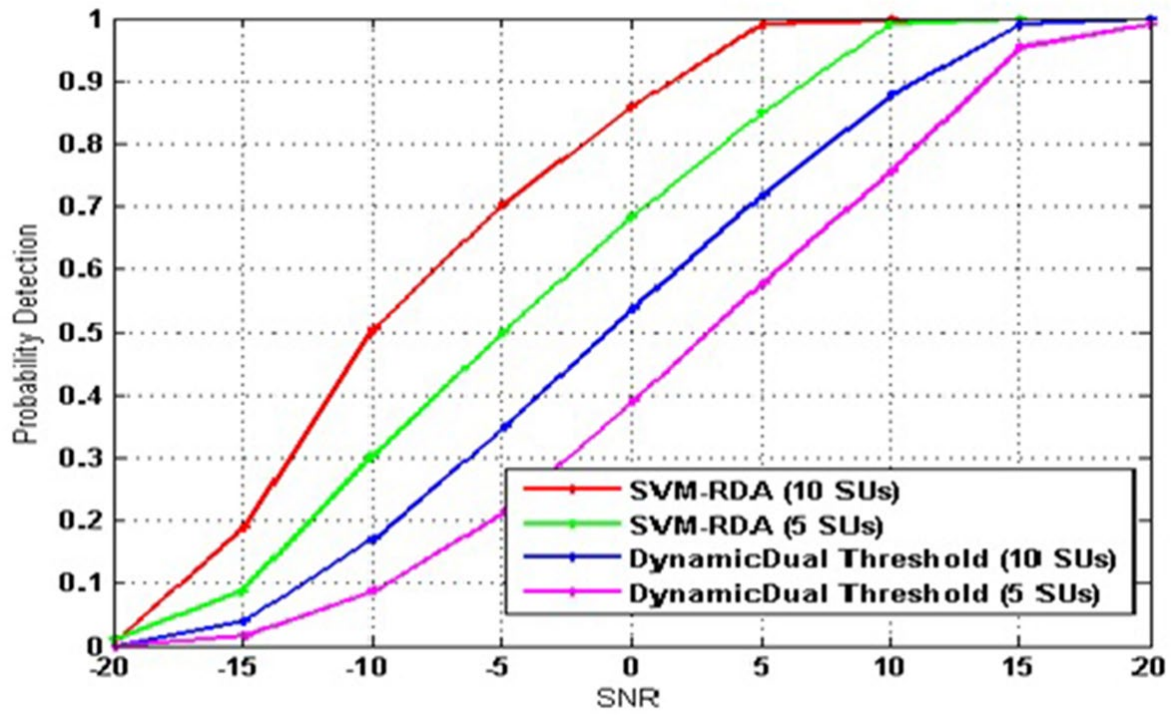
Probability of detection versus SNR at different cluster size.

For various SUs, Fig. 6 depicts the P_d_ related to the proposed algorithm (SVM–RDA) and dynamic dual-threshold model as an SNR function^38^. The SNR is between − 20 dB and 20 dB. As anticipated, P_d_ rises with SNR. P_d_ is less than 20% for SNR values below − 5 dB for 5 SUs and 10 SUs dynamic dual-threshold models. But, P_d_ is less than 70% for SNR values below − 5 dB for 5 SUs and 10 SUs for our proposed model (SVM–RDA). At SNR = 5 dB, the P_d_ is 100% for our proposed algorithm with 10 SU.

Furthermore, in both instances, P_d_ of 10 SUs is greater than P_d_ of 5 SUs. The probability of detection related to dynamic dual-threshold with 5 SU and 10 SU becomes very close to 20 dB SNR, and for a proposed model, it is SNR = 13 dB. So, number of cooperating SUs grows and detection performance improves.

**Figure 7:**
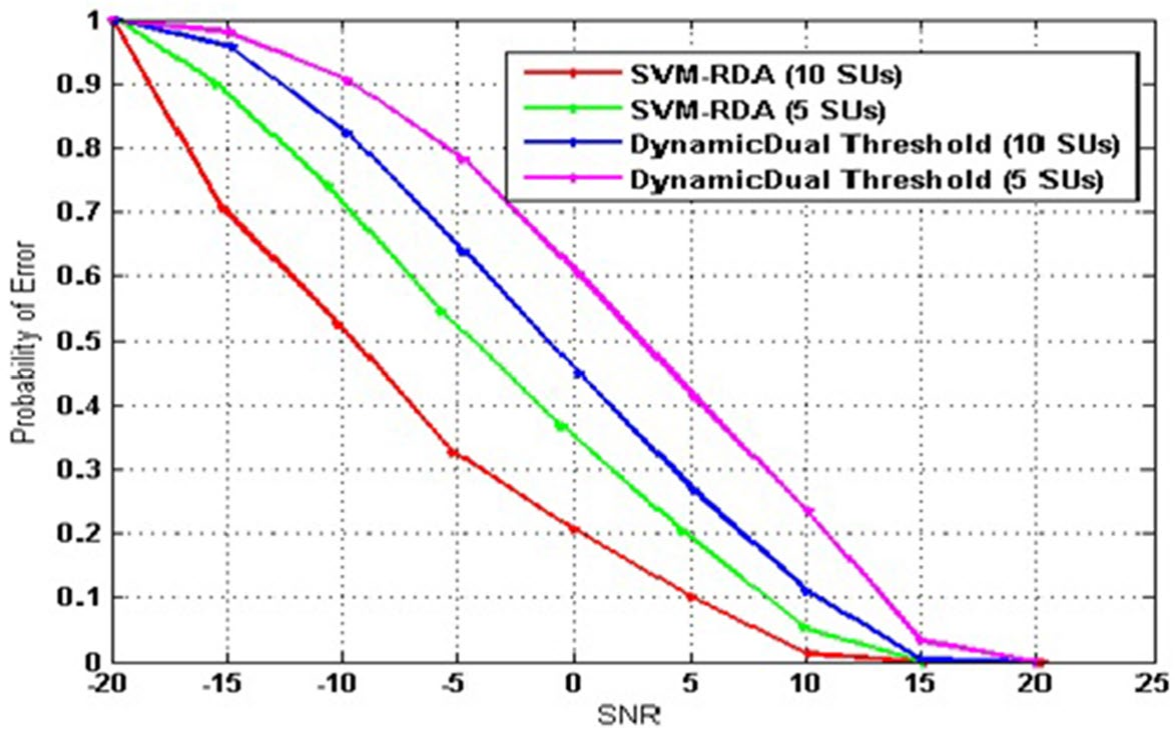
Probability of error versus SNR at different cluster size.

Figure 7 describes the P_e_ related to proposed algorithm (SVM–RDA) and dynamic dual-threshold model as an SNR function for different numbers of SUs. The probability of error reduces as the SNR increases. In addition, the P_e_ reduces as the number of cooperating SUs in cluster increases. P_e_ is high for 5 SUs and low for 10 SUs at high SNR, which can be explained by more cooperating users sensing the spectrum, resulting in lower P_e_. Thus, CSS-based clustering scheme reduces P_e_ by detecting unused spectrum with many users.

## Performance analysis

Table 1 demonstrates each optimization algorithm’s effectiveness [SVM–RDA, dynamic dual-threshold, Discover, Fuzzy energy detection, traditional energy detection, L1 Normalization for wavelet packet, Fast matching pursuit (for wavelet and wavelet packet domain) [FMP-W, FMP-WP] and threshold optimization matching pursuit for wavelet domain (TOMP-W)] in terms of P_d_, P_fa_, and different parameter values^36,38^.

**Table 1:** P_d_ and P_e_ values using SVM–RDA, dynamic threshold, for varying with SNR and occupied band.

Function	Optimisation algorithm	Probability of detection and Probability of error	
		P_d_	P_e_
Varying with SNR for 5 SU and 10 SU	SVM–RDA (10 SU) (at − 5 dB SNR)	0.7	0.32
	SVM–RDA (10 SU) (at 10 dB SNR)	1	0.02
	SVM–RDA (5 SU) (at − 5 dB SNR)	0.5	0.52
	SVM–RDA (5 SU) (at − 5 dB SNR)	0.99	0.05
	Dynamic Dual threshold (10 SU) (at − 5 dB SNR)	0.35	0.64
	Dynamic Dual threshold (10 SU) (at 10 dB SNR)	0.86	0.1
	Dynamic Dual threshold (5 SU) (at − 5 dB SNR)	0.21	0.79
	Dynamic Dual threshold (5 SU) (at 10 dB SNR)	0.75	0.24

At a − 5 dB SNR value, the proposed SVM–RDA has 46% and 9.8% more probability of detection than a dynamic dual-threshold for 5 SU and 10 SU, respectively. At 10 dB SNR, the proposed SVM–RDA has 24.24% and 14% higher detection probability than the dynamic dual-threshold for 5 SU and 10 SU, respectively. At the − 5 dB SNR value, the proposed SVM–RDA has 34.1% and 50% less probability of error than a dynamic dual-threshold for 5 SU and 10 SU, respectively. At a 10 dB SNR value, the proposed SVM–RDA has 79.16% and 18% less probability of error than a dynamic dual-threshold for 5 SU and 10 SU, respectively.

Compared to the current PSO-GSA, dynamic dual-threshold, Discover, Fuzzy energy detection, traditional energy detection, proposed algorithm is effective in spectrum sensing. It has obtained optimum energy consumption for spectrum sensing in CRN.

## Conclusion

It has been suggested that CSS can improve the sensing performance of a system when many SUs are monitoring the same band of interest. This article proposes a new approach to CSS using clustering. The SVM–RDA is a machine learning-based metaheuristic algorithm that is presented in this article. The combination of SVM and RDA has significantly improved the performance of SVM. The SVM–RDA has a good balance of local and global search capabilities. The outputs of the proposed technique are described and compared to the results of CSS with a mathematical model. The performance of the proposed technique is evaluated using the P_d_ and P_e_ metrics. The simulations show that cooperative radio spectrum sensing achieves excellent detection and low error rates. The proposed method has a P_d_ of greater than 99% and a P_fa_ false alarm probability of less than 1%, which is better than similar algorithms. The SVM–RDA approach outperforms current techniques in terms of spectrum sensing performance. The proposed method improves the performance of CRNs compared to the current approach due to its efficiency in spectrum sensing. The proposed model will be improved in future research by incorporating co-headship in the cluster instead of a single CH. This method distributes the burden of a single CH across multiple CHs. In the future, it is important to study how nodes in cognitive wireless sensor networks (CWSNs) work together to ensure detection performance and design more energy-efficient spectrum sensing technologies based on this.

## Data Availability

All data generated or analyzed during this study are included in this article.
